# Misclassification of recent HIV-1 seroconversion in sub-Saharan Africa using the sensitive/less sensitive technique

**DOI:** 10.1186/1743-422X-8-176

**Published:** 2011-04-17

**Authors:** Kwabena O Duedu, Anna A Hayford, Kwamena W Sagoe

**Affiliations:** 1 Clinical Virology Laboratory, Department of Microbiology, University of Ghana Medical School, Accra, Ghana; 2 National Public Health & Reference Laboratory, Ghana Health Service, Korle-Bu, Accra, Ghana

## Abstract

**Background:**

In resource-limited settings where HIV-1 is endemic, there is a need for simple, inexpensive but effective rapid methods for detecting recent infections and estimating incidence for the purposes of surveillance and management. We sort to determine possible reasons for reported misclassifications of recent HIV-1 seroconversion as determined with the S/LS assay in sub-Saharan Africa.

**Findings:**

We used the modified Determine HIV-1/2 sensitive/less sensitive method for determining recent HIV-1 seroconversion to determine recent infections among ELISA repeat HIV-1 positive samples from blood donors. Furthermore, HIV-1 seropositivity was confirmed using a line immunoassay and the results used to validate the performance of the modified Determine HIV-1/2 S/LS assay. The results confirmed reported misclassifications of recent HIV-1 seroconversion in sub-Saharan Africa. It was noted that, lack of confirmation of HIV-1 seropositivity in suspected cases of HIV-1 contributed to misclassifications.

**Conclusions:**

It was concluded that, with confirmation of HIV-1 seropositivity, the modified Determine HIV-1/2 S/LS assay will be a rapid and cost effective method for determining HIV-1 recent infections and estimating incidence in resource-limited settings. The need for detailed studies to validate simple methods for determining recent HIV-1 infections is emphasized.

## Findings

In resource-limited settings where HIV-1 is a burden, the need for reliable incidence surveillance data to help access performance of interventions as well as monitor transmission patterns cannot be overestimated. It is however unfortunate that such settings are coupled with challenges such as equipment and technical expertise. Standard assays, like the 3A11-LS for determining recent HIV infections may be impossible to use in these settings which therefore calls for alternative reliable but cheaper methods to determine recent HIV-1 seroconversion and estimate incidence. Improved laboratory methods for determining recent human immunodeficiency virus type 1 (HIV-1) seroconversion and estimating incidence have been widely reported [1-9]. Improvements in these methods help eliminate the use of longitudinal studies, back calculations and other cohort studies which are not only costly but also require consistent follow up of clients and repeated testing which is problematic and much difficult to perform in resource-poor settings [10]. Methods for determining recent HIV-1 infections in single serum samples include minipool (MP) nucleic acid amplification testing (NAT) and individual donation (ID) NAT, detection of p24 antigen and the sensitive/less sensitive (S/LS) method for detecting recent HIV-1 seroconversion [3]. NATs and p24 antigen testing are costly and require specific laboratory equipments to perform. A report on the cost-effectiveness of including MP or ID NAT and or p24 antigen testing to blood screening in Ghana found that it was highly costly to perform such tests [11]. The sensitive/less sensitive strategy however seems simpler and 'less costly' to be used in resource-limited settings. Detection of recent seroconversion can provide information on serologically undetected residual infections, monitor transmission trends in a community and also determine incidence of recent HIV-1 infection. Studies have been performed in some African countries using the S/LS EIA to determine recent HIV-1 seroconversion and estimate incidence [12-15]. It has however been reported that some of these assays give misleading results when used in Sub-Saharan Africa [16].

Recent HIV-1 seroconversion was determined among 76 blood donor plasma samples which tested repeatedly double reactive on the Genescreen Ultra HIV Ag-Ab ELISA which was used at the time of the study for routine HIV screening at the National Public Health & Reference Laboratory, Accra, Ghana. Of the 76 ELISA repeat reactive samples, 41 tested repeatedly reactive on the Determine™ HIV-1/2 (Abbott Laboratories, Abbott Park, IL) rapid test. These plasma samples (n = 41) were subsequently taken through the modified Determine S/LS (Det-S/LS) protocol as described elsewhere [8]. The protocol classified 23 as recent seroconversions and the remaining 18 as longstanding seroconversions. Due to previous reports of misclassifications, we further performed the INNO-LIA™ confirmatory assay to ascertain the HIV-1 antibody seropositivity on all the initial 76 ELISA double repeat reactive samples. After confirmation, only one of the 23 recent seroconversion samples turned out to be seropositive for HIV-1. It was thus concluded that only this was a true recent HIV-1 seroconversion. All the 35 negative samples as determined by the Determine™ HIV-1/2 rapid test initially were also negative by INNO-LIA™. All the longstanding seroconversions as determined by the modified Det-S/LS protocol were confirmed as seropositive for HIV-1. On the INNO-LIA™ strip, the one recent seroconversion showed intense (3+) bands for antibodies to five HIV-1 proteins (sgp120, gp41, p31, p24, & p17).

For blood donor screening, the use of a very sensitive assay is recommended. These assays though having high sensitivities are not very specific as observed in this study. In the absence of PCR, INNO-LIA™ is a good alternative gold method for confirming HIV-1 and HIV-2 seropositivity. This is employed in many resource-limited settings where PCR is not available. In this study, the existing systems were used to troubleshoot a local problem. It was observed that, screening samples with the Det-S/LS assay without confirmation was likely to result in an overestimation of recent HIV seroconversion. Any calculations therefore using the misclassified recent data would result in overestimation of incidence of HIV-1 infection. Though fourteen (14) of the initial recent seroconversions were indeterminate on INNO-LIA™, the classification algorithm for recent infections by INNO-LIA as reported by Schupbach and his colleagues [17] could not be used due to absence of clinical information on the blood donor samples used. This classification however is questionable by our results as recent HIV seroconversion did not correspond to faint banding patterns on INNO-LIA™ as reported. We will therefore like to stress that, incidence estimates calculated with recent seroconversion data obtained using S/LS and other antibody based recent seroconversion algorithms are likely to be overestimated if HIV seropositivity is not confirmed.

**Figure 1:**
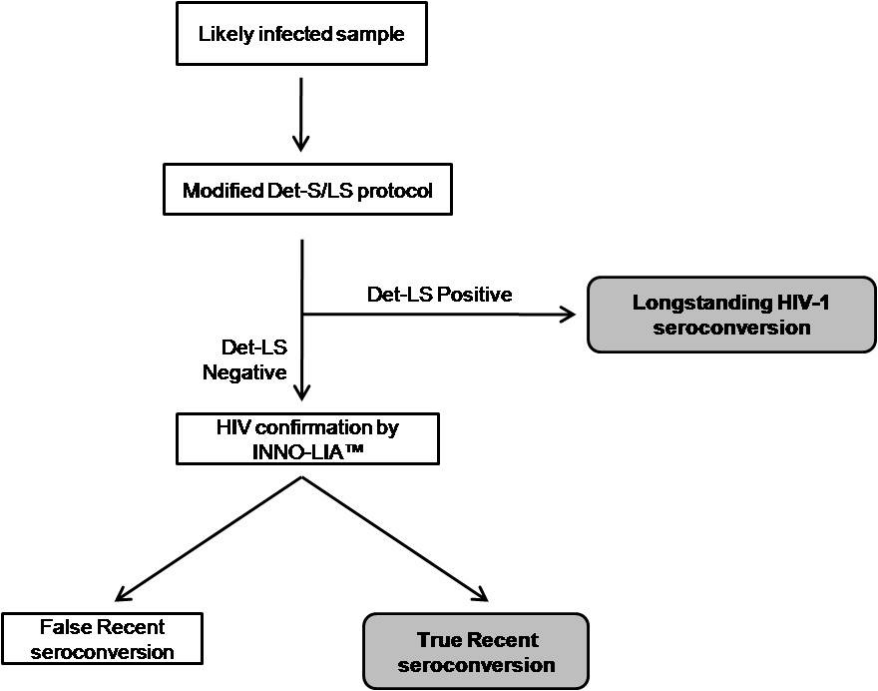
**Algorithm for determining recent HIV-1 seroconversion using the modified Determine sensitive/less sensitive protocol**.

Our results also indicate that, the modified rapid tests may be a good alternative for determining recent HIV-1 seroconversion and estimating incidence in resource-limited settings. There is therefore a need extensive work into this area of research. Since INNO-LIA™ is used as a confirmatory test for HIV in Ghana and other settings, there is a need for further studies to characterize seroconversion status of individuals whose antibody status are indeterminate on INNO-LIA™ and clearly define an algorithm for classifying recent seroconversion as suggested [17]. By this study we propose a simple cost-effective algorithm for determining recent HIV-1 seroconversion in resource limited settings (figure 1). The likely positive samples are taken through the Det-S/LS protocol as described by Soroka et al [8]. Suspected recent seroconversions are subsequently confirmed for their HIV-1 seropositivity using INNO-LIA™. The number of tests for INNO-LIA™ at this stage thus will be reduced as compared to performing INNO-LIA™ from step 1. In bigger systematic studies, persons classified by this algorithm as false recent seroconvertions may be followed up or a PCR performed where the facility exists to ascertain whether they are really negative for HIV-1 or not.

## Competing interests

The authors declare that they have no competing interests.

## Authors' contributions

KOD and KWCS conceptualized the study. KOD and AAH obtained samples and carried out the tests. KOD drafted the manuscript. KWCS reviewed the manuscript. All authors read and approved the manuscript.

## References

[B1] Chawla A Murphy G Donnelly C Booth CL Johnson M Parry JV Phillips A Geretti AM Human immunodeficiency virus (HIV) antibody avidity testing to identify recent infection in newly diagnosed HIV type 1 (HIV-1)-seropositive persons infected with diverse HIV-1 subtypes J Clin Microbiol 2007 45 415 420 10.1128/JCM.01879-06 17151211 PMC1829080

[B2] Constantine NT Sill AM Jack N Kreisel K Edwards J Cafarella T Smith H Bartholomew C Cleghorn FR Blattner WA Improved classification of recent HIV-1 infection by employing a two-stage sensitive/less-sensitive test strategy J Acquir Immune Defic Syndr 2003 32 94 103 10.1097/00126334-200301010-00014 12514420

[B3] Janssen RS Satten GA Stramer SL Rawal BD O'Brien TR Weiblen BJ Hecht FM Jack N Cleghorn FR Kahn JO New testing strategy to detect early HIV-1 infection for use in incidence estimates and for clinical and prevention purposes JAMA 1998 280 42 48 10.1001/jama.280.1.42 9660362

[B4] Parekh BS Kennedy MS Dobbs T Pau CP Byers R Green T Hu DJ Vanichseni S Young NL Choopanya K Quantitative detection of increasing HIV type 1 antibodies after seroconversion: a simple assay for detecting recent HIV infection and estimating incidence AIDS Res Hum Retroviruses 2002 18 295 307 10.1089/088922202753472874 11860677

[B5] Parekh BS McDougal JS Application of laboratory methods for estimation of HIV-1 incidence Indian J Med Res 2005 121 510 518 15817960

[B6] Rawal BD Degula A Lebedeva L Janssen RS Hecht FM Sheppard HW Busch MP Development of a new less-sensitive enzyme immunoassay for detection of early HIV-1 infection J Acquir Immune Defic Syndr 2003 33 349 355 10.1097/00126334-200307010-00009 12843746

[B7] Sill AM Kreisel K Deeds BG Wilson CM Constantine NT Peralta L Calibration and validation of an oral fluid-based sensitive/less-sensitive assay to distinguish recent from established HIV-1 infections J Clin Lab Anal 2007 21 40 45 10.1002/jcla.20144 17245763 PMC6649113

[B8] Soroka SD Granade TC Candal D Parekh BS Modification of rapid human immunodeficiency virus (HIV) antibody assay protocols for detecting recent HIV seroconversion Clin Diagn Lab Immunol 2005 12 918 921 16085908 10.1128/CDLI.12.8.918-921.2005 PMC1182197

[B9] Suligoi B Galli C Massi M Di Sora F Sciandra M Pezzotti P Recchia O Montella F Sinicco A Rezza G Precision and accuracy of a procedure for detecting recent human immunodeficiency virus infections by calculating the antibody avidity index by an automated immunoassay-based method J Clin Microbiol 2002 40 4015 4020 10.1128/JCM.40.11.4015-4020.2002 12409368 PMC139667

[B10] Rutherford GW Schwarcz SK McFarland W Surveillance for incident HIV infection: new technology and new opportunities J Acquir Immune Defic Syndr 2000 25 Suppl 2 S115 119 10.1097/00042560-200012152-00005 11256731

[B11] van Hulst M Sagoe KW Vermande JE van der Schaaf IP van der Tuuk Adriani WP Torpey K Ansah J Mingle JA Smit Sibinga CT Postma MJ Cost-effectiveness of HIV screening of blood donations in Accra (Ghana) Value Health 2008 11 809 819 10.1111/j.1524-4733.2008.00337.x 18489518

[B12] Karita E Price M Hunter E Chomba E Allen S Fei L Kamali A Sanders EJ Anzala O Katende M Ketter N Investigating the utility of the HIV-1 BED capture enzyme immunoassay using cross-sectional and longitudinal seroconverter specimens from Africa AIDS 2007 21 403 408 10.1097/QAD.0b013e32801481b7 17301558

[B13] Moyo S Bodika SM Wester WC Roels TH Mlotshwa BC Mphoyakgosi K Negussie T Bussmann H Bile E Seipone K Estimation Of HIV incidence in among pregnant women attending antenatal clinics In Botswana in 2005 using serological test for recent seroconversion4th IAS Conference on HIV pathogenesis, treatment and prevention 2007 Sydney, Australia: International AIDS Society

[B14] Gouws E Williams BG Sheppard HW Enge B Karim SA High incidence of HIV-1 in South Africa using a standardized algorithm for recent HIV seroconversion J Acquir Immune Defic Syndr 2002 29 531 535 11981371 10.1097/00126334-200204150-00015

[B15] Riviello ED Sterling TR Shepherd B Fantan T Makhema J HIV in the workplace in Botswana: incidence, prevalence, and disease severity AIDS Res Hum Retroviruses 2007 23 1453 1460 10.1089/aid.2007.0132 18160001

[B16] Sakarovitch C Rouet F Murphy G Minga AK Alioum A Dabis F Costagliola D Salamon R Parry JV Barin F Do tests devised to detect recent HIV-1 infection provide reliable estimates of incidence in Africa? J Acquir Immune Defic Syndr 2007 45 115 122 10.1097/QAI.0b013e318050d277 17460475

[B17] Schupbach J Gebhardt MD Tomasik Z Niederhauser C Yerly S Burgisser P Matter L Gorgievski M Dubs R Schultze D Assessment of recent HIV-1 infection by a line immunoassay for HIV-1/2 confirmation PLoS Med 2007 4 e343 10.1371/journal.pmed.0040343 18052604 PMC2100138

